# Can Adhering to Clinical Communication Guidelines Promote Trust, Liking and Respect for General Practitioners and Willingness to Discuss Depression? A Theory-Based Test

**DOI:** 10.3390/bs16050668

**Published:** 2026-04-28

**Authors:** Kiara Crawford, Andrew Prestwich

**Affiliations:** School of Psychology, University of Leeds, Leeds LS2 9JT, UK; kiaracrawford13@gmail.com

**Keywords:** Morality–Agency–Communion model, depression, respect, liking, trust, theory, general practitioner

## Abstract

Despite its prevalence, individuals can be reluctant to discuss depression with their general practitioners (GPs). Effective communication strategies can help but their relative impact on patients’ willingness to discuss their mental health and the underpinning theoretical mechanisms is unclear. Using the Morality–Agency–Communion (MAC) model as the theoretical basis, a UK-based online sample (*N* = 329) rated their respect, liking and trust for GPs (MAC model outcomes), along with their perceived competence, warmth, morality and assertiveness (MAC model trait mechanisms) and willingness to discuss depression (downstream outcome) before and after reading six hypothetical scenarios based on UK communication guidelines. Compared to baseline, following each recommendation improved all outcomes. Liking was the most consistent mediator on willingness to discuss depression; trust, and to a lesser extent, respect, also played a role. Trait mechanisms differed by outcome: warmth was the most robust mediator for liking; for trust, competence; for respect, morality was key. Of the six communication approaches, allowing patients time to describe and discuss any problems or concerns was the most impactful. Consistent with the MAC model, this study suggests that following communication guidelines can improve how much GPs are seen as moral, warm and competent and, in turn, how much they are respected, liked and trusted. GPs could utilise these approaches more consistently to increase how much they are liked and trusted as well as their patients’ willingness to discuss depression within their consultations.

## 1. Introduction

Mental health difficulties represent a major public health concern, affecting individuals’ thoughts, emotions and behaviours (World Health Organization, 2022). Depression is particularly prevalent, impacting an estimated 280 million people globally (World Health Organization, 2023). Common symptoms include continuous low mood, hopelessness, diminished self-esteem and a loss of interest in once pleasurable activities (NHS, 2023). General practitioners (GPs) are frequently the main point of formal contact for individuals experiencing depressive symptoms (Clery et al., 2025). In the United Kingdom, GPs conduct around 30 consultations per day, with many doing far more (Pulse, 2024), with approximately 40% involving mental health concerns (Mind, 2018). Thus, there is a growing demand for GPs to communicate in ways that foster open discussion, which aids in early assessment, recognition and intervention (Royal College of General Practitioners [RCGP], 2017).

The National Institute for Health and Care Excellence (NICE) provides practitioners with guidance on effective communication in mental health contexts, emphasising clarity, emotional support, exploring patients’ concerns and shared decision-making (NICE, 2011). Research indicates that effective clinician communication improves how they are perceived (e.g., Greene & Ramos, 2021), as well as treatment adherence (Thompson & McCabe, 2012), patient satisfaction (e.g., Chen et al., 2025), emotional health outcomes (Riedl & Schüßler, 2017) and patients’ willingness to talk about their emotional concerns (Parker et al., 2020). Despite this, patients often hesitate to disclose depression to GPs with stigma, fear of judgment, confidentiality and the desire for self-reliance being key barriers (Gulliver et al., 2010; Clement et al., 2015). Time pressures in primary care further complicate these interactions, having a negative impact on doctor–patient relationships and increasing the risk of missed opportunities for early intervention and increasing the need for follow-up care (Dugdale et al., 1999; Freedman et al., 2021).

### 1.1. Using Theory to Examine the Role of Health Practitioner Communication on Willingness to Discuss Depression

Theory, by outlining a set of concepts, their interrelationships and a set of propositions, provides a systematic way to understand events and outcomes (Glanz & Rimer, 2005). It provides a framework to test hypotheses and gather evidence as well as identifying constructs (or mechanisms of action) that can be targeted by interventions to change behaviour and can provide insights regarding why behavioural interventions may work or fail (Prestwich et al., 2015; Prestwich et al., 2024). While theories have often been tested using correlational methods to establish the relationships between constructs, there have been calls to test theories experimentally (e.g., Sheeran et al., 2017) and to consider not only whether an intervention can change theoretical constructs but also how much changing theoretical constructs leads to changes in behaviour (Riddle & Science of Behavior Change Working Group, 2015).

Respect, liking and trust are fundamental to effective GP–patient relationships. Respect involves holding people (including GPs and patients) in high regard (Lalljee et al., 2009). Trust reflects allowing oneself to be vulnerable to others believing they have one’s best interests at heart (e.g., Mayer et al., 1995; Hall et al., 2001; Lerch et al., 2024), while liking reflects preference (e.g., for one GP over another) or fondness for other people. These constructs contribute to subjective health outcomes (e.g., Birkhäuer et al., 2017), the likelihood of seeking care (e.g., Kannan & Veazie, 2014), satisfaction with care (e.g., Williams & Calnan, 1991), treatment adherence (e.g., Thom et al., 1999) and feelings of a deeper relationship (Thomas et al., 2023). Consequently, respect, liking and trust of GPs represent important mechanisms of action, or targets for interventions, to promote patient behaviours such as treatment adherence and discussing sensitive topics such as one’s mental health. Identifying the factors that influence respect, liking and trust, and testing the effect of changing these on patient outcomes, are thus important issues to address.

Previous research has suggested that agentic traits (those that help individuals to get ahead) are particularly important for respect while communal traits (those that help individuals to get along with others) are particularly important for liking (e.g., Wojciszke et al., 2009). However, more recent research has indicated that communal traits can be subdivided into morality and warmth and agentic traits into competence and assertiveness (e.g., Abele et al., 2016) and the type of communal trait can influence their impact on respect and liking. The Morality–Agency–Communion (MAC) model (Prestwich et al., 2021) builds on this division and proposes that warmth primarily fosters liking, competence and assertiveness foster respect, with morality being important for both respect and liking (see also Hartley et al., 2016). The model has received support in health (e.g., Prestwich et al., 2025b) and non-health contexts (e.g., Prestwich, 2024).

The MAC model was recently used by Prestwich et al. (2025a) to test the effects of adhering to, or violating, specific communication guidelines on patients’ respect, liking and trust of GPs as well as patients’ commitment to following treatment advice. In the context of GPs’ behaviour, morality reflects ethical behaviour, acting in the patient’s best interests and respecting patient autonomy (Gillon, 1994, 2015), competence reflects clinical skill, knowledge, motivation and effectiveness with appropriateness (Howe et al., 2019; General Medical Council [GMC], 2025; Spitzberg, 1983), warmth is expressed through empathy and engagement (Howe et al., 2019), and assertiveness involves communicating clearly and confidently. Consistent with the MAC model, they found that competence (and to a lesser extent, morality) mediated the effect of communication on respect; warmth (and to a lesser extent, morality) were mediators for liking. In the MAC model extended to trust, it was hypothesised that competence, morality, warmth and assertiveness would play a role. For trust, competence was the most robust mediator, with some evidence also for morality and warmth.

As well as testing the effect of hypothetical interventions (adhering to, or violating, communication guidelines) on mechanisms of action (MAC constructs of competence, assertiveness, morality and warmth) and examining the extent that changes in these mechanisms corresponded with changes in respect, liking and trust; they found that trust, and to a lesser degree, respect mediated the effect of the communication strategies on commitment to following treatment advice. Further, by testing multiple specific communication strategies, they provided a competitive test of engagement pathways between the intervention and the target/mechanism of action; something that has been identified as being infrequent in health behaviour change literature (Sheeran et al., 2017). Of the four tested communication strategies, summarising information at the end of the consultation and checking that the patient understands the most important information was the approach that impacted the outcomes the most.

Despite providing some support for the MAC model, some findings reported by Prestwich et al. (2025a) were not always wholly consistent with the MAC model. First, in some analyses, competence unexpectedly mediated liking. However, in the context of the scenarios describing GPs as communicating in a particular way, participants could base perceptions of GP competence on GPs’ interpersonal competence, which is likely important for liking. Second, morality was a less reliable mediator than predicted (competence was more consistent for respect; warmth was more consistent for liking). Third, there was some support that warmth mediated effects on respect (although these disappeared after controlling for liking). The superordinate position of the GP and subordinate position of the patient could elevate the impact of warmth on respect (as well as trust and liking); Oleszkiewicz and Lachowicz-Tabaczek (2016) reported that warmth shown by supervisors compared to subordinates led to greater levels of liking, trust and respect. Such findings suggest that some tenets of the MAC model may be moderated by the status of the evaluated person and the observer. Fourth, despite the MAC model stating that assertiveness can be important for building respect (and, in the extended model, trust) there was limited support. However, researchers have argued that assertiveness must be calibrated carefully, as excessive or improperly applied assertiveness can damage the doctor–patient relationship (Gutgeld-Dror et al., 2024). These findings suggest that the MAC framework requires further testing in the context of doctor–patient interactions.

The current study extends previous work by examining, for the first time in the context of applying the MAC model to provide mental health support, the impact of hypothetical communication strategies on perceptions of GPs and willingness to discuss mental health. Specifically, a theory-based approach is taken to test the effect of different hypothetical communication strategies on MAC-model mediators (competence, morality, warmth and assertiveness) and outcomes (respect and liking, plus trust) and the downstream outcome of willingness to discuss depression. As well as testing the engagement pathway (effect of interventions on mediators), it tests the validation pathway (whether changes in mediators correspond with changes in outcomes) consistent with an experimental medicine approach (Riddle & Science of Behavior Change Working Group, 2015). Given infrequent competitive tests of intervention strategies on health-related outcomes (Sheeran et al., 2017), a secondary aim was to present competitive tests of different communication strategies. In addition, we explored whether the effect of different types of communication on the outcome variables was moderated by previous feelings of depression.

### 1.2. Hypotheses

The following preregistered hypotheses were tested:

**H1:**  *Compared to baseline, adhering to NICE-recommended communication strategies will increase (H1a) respect, (H1b) liking, (H1c) trust that participants have for GPs and (H1d) willingness to discuss depression with a GP. In addition, they will rate the GPs as more (H1e) competent, (H1f) moral, (H1g) assertive and (H1h) warm compared to baseline.*

**H2:**  *Increased willingness to discuss depression following GP communication will be mediated by increases in (H2a) respect, (H2b) liking and (H2c) trust.*

**H3:**  *Increases in respect will be mediated by increases in (H3a) competence, (H3b) morality and (H3c) assertiveness.*

**H4:**  *Increases in trust will be mediated by increases in (H4a) competence, (H4b) morality, (H4c) assertiveness and (H4d) warmth.*

**H5:**  *Increases in liking will be mediated by increases in (H5a) warmth and (H5b) morality.*

## 2. Materials and Methods

The study was pre-registered (https://aspredicted.org/aa9a4d.pdf (accessed on 18 February 2025)) and received ethical approval from the School of Psychology, University of Leeds, Research Ethics Committee {reference: PSCETHS-1413; date of approval: 4 March 2025]. Study materials, data and syntax are available on the OSF (https://osf.io/x3qs7/overview?view_only=91104ec58f8744ec9d2113af67cbc076 (accessed on 18 February 2025)).

### 2.1. Participants

To detect a minimally meaningful effect (*d* = 0.20) with 90% power at *p* < 0.01 (one-tailed) in a repeated measures t-test requires 329 participants. Eligibility criteria required participants to be aged 18 or older, UK nationals, fluent in English and resident in the UK. Participants (*N* = 399) were recruited via Prolific (https://www.prolific.com). Consistent with the pre-registration, 70 participants were excluded (*n* = 18 completed the survey in less than 2 min and 15 s, of which 7 also failed the attention check; *n* = 52 did not respond too quickly but failed the attention check). The final sample (*n* = 329; gender described as female/woman: 215; male: 110; non-binary: 3) were aged 18–80 years (*M* = 41.31 years, *SD* = 13.76), most often reported their ethnicity as White (e.g., White and White British *n* = 246), comprised of 39 who responded that they were a student and none that they were a GP. Regarding mental health history, 226 participants reported prior experience of depression, 99 reported none, and 4 preferred not to say. Participants were reimbursed GBP 0.50 for their time.

### 2.2. Design

Participants responded to baseline and 6 hypothetical GP scenarios relating to adhering to NICE-recommended communication strategies in a within-subjects design. Following completion of the baseline measures, the order of the GP scenarios was randomised. All participants were presented with all scenarios and all measures. An attention check item (‘Please respond 3 on the rating scale for this item’) was embedded within the rating scale items.

### 2.3. Scenarios

Six GP communication scenarios were developed from NICE guidelines for mental health consultations (NICE, 2011):

**GP 1**—GP 1 always asks patients how they wish to be addressed and addresses them by their preferred name and title.
**GP 2**—GP 2 always explains any clinical or unfamiliar language.
**GP 3**—GP 3 always provides patients with information about their different treatment options and their side effects and discusses these with their patients.
**GP 4**—GP 4 always ensures there is enough time for patients to describe and discuss any problems or concerns that they may have.
**GP 5**—GP 5 always ensures that all discussions take place in settings in which patients’ confidentiality, privacy, and dignity are maintained.
**GP 6**—GP 6 always allows enough time towards the end of the appointment for summarising the conclusions of the assessment and for discussion that allows questions and answers.

### 2.4. Measures

All constructs were measured using single items along a series of 1–7 rating scales ranging from strongly disagree (1) to strongly agree (7). A total of 8 constructs were assessed: respect (I respect GPs), trust (I trust GPs), liking (I like GPs), competence (GPs are competent), warmth (GPs are warm), assertiveness (GPs are assertive), morality (GPs are moral) and willingness to discuss depression (If I were to experience symptoms of depression (e.g., continuous low mood, feeling hopeless), I would be willing to discuss these with GPs). Demographic information (age, gender, ethnicity, prior depression experience, whether participants were a GP and student status) was also collected.

### 2.5. Procedure

The study was completed online via Qualtrics. After providing informed consent, demographic items were completed, then baseline ratings of respect, trust, liking of GPs, and perceptions of their warmth, competence, morality and assertiveness, and willingness to discuss depression with GPs. Participants repeated the same ratings for 6 hypothetical GPs each demonstrating a distinct NICE-recommended communication strategy for mental health consultations (NICE, 2011). Participants were debriefed after completion of the questionnaire. The median average completion time for the final sample (*n* = 329) was 4 min and 27 s.

### 2.6. Analyses

Two-way within-subjects ANOVA (scenario, 7 levels: baseline, communication 1–6 x measure, 3 levels: respect, liking and trust), followed-up with one-way within-subjects ANOVA for each measure and repeated measures t-tests (equivalent to simple contrasts), tested H1a to H1h. Where the assumption of sphericity was violated either the Greenhouse–Geisser (ε < 0.75) or Huynh–Feldt corrections (ε > 0.75) were applied. For exploratory comparisons between different types of GP communications (not pre-registered), Bonferroni corrections were applied. 2 × 2 within-subjects ANOVAs were conducted to compare changes in respect vs. liking, respect vs. trust and liking vs. trust for each GP communication vs. baseline. Equivalent analyses were conducted to compare changes across competence, assertiveness, morality and warmth. The main analyses were repeated with depression added as a moderator to examine whether depression moderated the key effects. Within-subjects mediation tested H2 to H5 using the SPSS MEMORE macro version 2.1 (Montoya & Hayes, 2017).

## 3. Results

### 3.1. Respect, Liking and Trust

A main effect of measure, *F*(1.91, 626.25) = 107.13, *p* < 0.001, η_p_^2^ = 0.25, indicated GPs were rated differently across measures. On average, GPs were respected more than trusted (*p* < 0.001) or liked (*p* < 0.001) and trusted more than liked (*p* < 0.001).

A significant main effect of GP communication indicated that GP evaluations (across respect, liking and trust) differed across scenarios, *F*(4.73, 1552.68) = 72.63, *p* < 0.001, η_p_^2^ = 0.18. Compared with baseline, five GPs were evaluated more positively overall (GPs 2–6: *p* < 0.001). GP 1 was not evaluated more favourably than baseline (*p* = 0.05) and was rated lower than all other GPs (GP 2, *p* = 0.007, and GPs 3–6, *p* < 0.001). GP 4 was evaluated most favourably, exceeding GPs 1 and GP 2 (*p* < 0.001), GP 3 (*p* = 0.009), and GP 6 (*p* = 0.003). There were no significant differences between GPs 3, 5 and 6 (*p* = 1).

**Figure 1:**
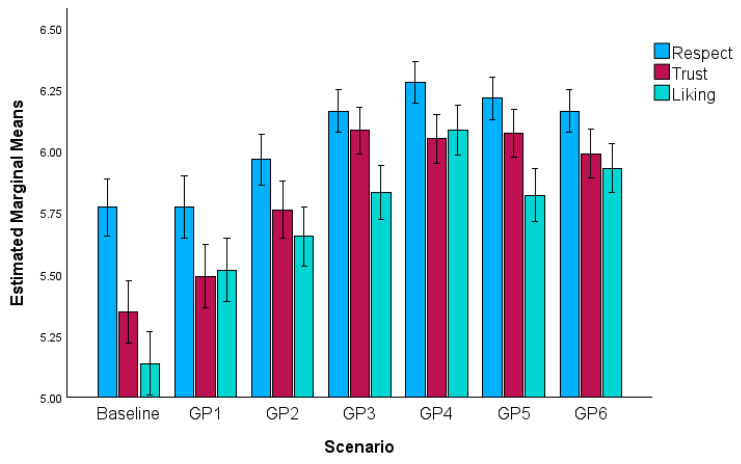
Respect, trust and liking across baseline and GP communication scenarios (95% confidence intervals).

A significant GP communication x measure interaction showed that the effect of GP communication on ratings varied by measure, *F*(10.42, 3418.27) = 16.00, *p* < 0.001. GPs 1–6 increased trust (GP 1, *p* = 0.006; GPs 2–6, all *p* < 0.001) and liking (all *p* < 0.001) more than respect from baseline. GP 1 (*p* < 0.001), GP 4 (*p* < 0.001) and GP 6 (*p* = 0.007) improved liking more than trust but GP 2 (*p* = 0.07), GP 3 (*p* = 0.44) and GP 5 (*p* = 0.43) increased trust and liking similarly from baseline (see Figure 1 and Table S1).

Aside from GP 1 (*p* = 1), all GP communications increased respect relative to baseline (all *p* < 0.001) consistent with H1a. GP 4 was respected more than the other GPs (GP 1–2, *p* < 0.001; GP 3, *p* = 0.03; GP 6, *p* = 0.01) except GP 5 (*p* = 1). Supporting H1b, all GP communications increased liking from baseline (all *p* < 0.001). GP 4 was liked more than all other GPs (GPs 1–3 and 5, *p* < 0.001; GP 6, *p* = 0.004). GP 1 (*p* = 0.02) and GPs 2–6 (all *p* < 0.001) increased trust from baseline (supporting H1c). GPs 3–6 were similarly effective in increasing trust (all *p* > 0.05) and all outperformed GPs 1–2 (all *p* < 0.001).

### 3.2. Willingness to Discuss Depression

A main effect of communication indicated that willingness to discuss depression differed across the GP scenarios, *F*(5.10, 1674.02) = 56.61, *p* < 0.001. Supporting H1d, all six strategies increased willingness relative to baseline (*p* < 0.001). GP 4 produced the highest willingness ratings, exceeding all other conditions (GP 5, *p* = 0.002; GPs 1–3 and 6, *p* < 0.001), and GP 1 the lowest ratings, significantly below GPs 3–6 (*p* < 0.001). There was no significant difference between GPs 1 and 2 (*p* = 0.74), and between GPs 3, 5 and 6 (all *p* = 1).

### 3.3. Perceived Competence, Warmth, Morality and Assertiveness

A main effect indicated ratings varied across measure type, *F*(1.81, 594.51) = 126.46, *p* < 0.001. GPs were rated highest on competence, followed by morality, warmth and assertiveness (all *p* < 0.001).

A main effect of communication indicated that GPs were evaluated differently, *F*(5.02, 1646.58) = 78.56, *p* < 0.001. Compared to baseline, GPs 1–6 were rated more favourably (all *p* < 0.001). GPs 1 and 2 were rated similarly (*p* = 0.32) and less favourably than GPs 3–6 (all *p* < 0.001). There was no significant difference between GPs 3 and 6 (*p* = 1).

**Figure 2:**
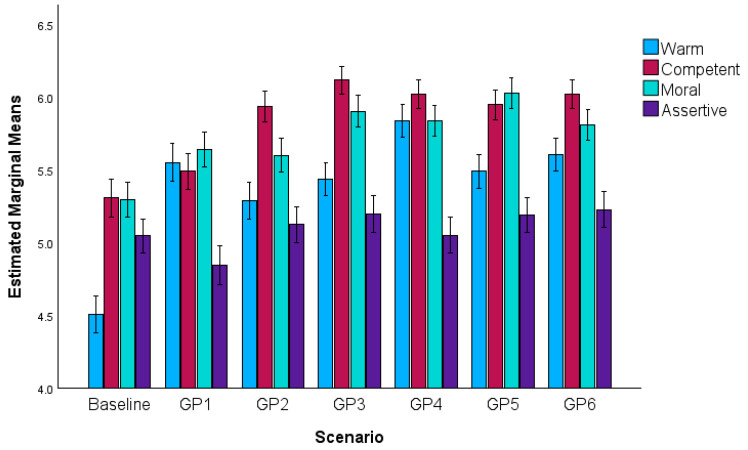
Warmth, competence, morality, and assertiveness across baseline and GP communication scenarios (95% confidence intervals).

A significant communication x measure interaction suggested that the impact of different GP communications varied across the four measures, *F*(13.10, 4297.93) = 40.90, *p* < 0.001. Follow-up 2 (scenario vs. baseline) × 2 (measure) ANOVAs indicated that each scenario increased perceptions of GP warmth, competence and morality more than assertiveness (all *p* < 0.001, except GP 2 for morality, *p* = 0.002); increased warmth more than morality (all *p* < 0.001) and generally warmth more than competence (all *p* < 0.001; except for GP 2 where the increases, while statistically different, *p* = 0.034, were similar in size *d* = 0.60 vs. *d* = 0.58; and GP 3, *p* = 0.08). Moreover, perceptions of competence generally increased more than morality (GP 2, GP 3 and GP 6, *p* < 0.001; GP 4, *p* = 0.003) though GP 1 increased morality more than competence (*p* = 0.009) and there were no differences for GP 5 (*p* = 0.10) (see Figure 2 and Table S1).

Relative to baseline, each GP communication significantly increased competence (all *p* < 0.001, except GP 1, *p* = 0.005 without correction, *p* = 0.097 with Bonferroni correction); supporting H1e, with GP 3 significantly outperforming GP 1–2 (both *p* < 0.001) and GP 5 (*p* = 0.002) but not GP 4 (*p* = 0.44) or GP 6 (*p* = 0.22)), morality (all *p* < 0.001; supporting H1f; GP 5 was rated higher on morality than all other GPs (all *p* < 0.001) except GP 3 (*p* = 0.14)) and warmth (all *p* < 0.001; supporting H1h, with GP 4 rated higher than all other GPs, all *p* < 0.001).

The GP communication had more mixed effects on assertiveness (providing mixed support for H1g): while GP 3 (*p* = 0.02), GP 5 (*p* = 0.02) and GP 6 (*p* = 0.006) increased assertiveness relative to baseline, these were non-significant with Bonferroni corrections (see Table S1), GP 1 decreased assertiveness (*p* = 0.002) and GP 2 (*p* = 0.25) and GP 4 (*p* = 0.96) did not promote assertiveness.

### 3.4. Moderation by Depression History

Repeating the main ANOVAs, adding in previous experience of depression as a moderator, indicated that previous experience of depression did not moderate any of the main effects or interactions (all *p* > 0.05; ANOVA 1: perceptions of respect, liking and trust of GPs; ANOVA 2: willingness to discuss depression; ANOVA 3: perceptions of GP warmth, competence, morality and assertiveness).

### 3.5. Mediation Analyses

#### 3.5.1. Willingness to Discuss Depression

In all single-mediator models, trust and liking significantly mediated the relationship between GP communication (vs. baseline) and willingness to discuss depression across all conditions (see Table S2). Respect was a significant mediator in all but one single-mediator model (non-significant for GP 1). Liking was a robust and consistent mediator across all multiple-mediator models (supporting H2b); trust was a significant mediator for all but one (GP 1) multiple-mediator model (providing relatively strong support for H2c). Respect was significant in just one of the multiple mediator models (GP 4) (providing limited support for H2a).

#### 3.5.2. Respect

Morality was the most robust mediator of the relationship between GP communication scenario (vs. baseline) and respect given its significance in most multiple mediator models (including 4 out of 6 models that included liking and trust; consistent with H3b). There was some, but less consistent evidence, support for the mediating role of competence (supporting H3a) and warmth. There was no support for the mediating role of assertiveness (see Table S3; providing no support for H3c).

#### 3.5.3. Trust

Consistent with H4a, competence was a robust mediator of the relationship between GP communication and trust of GPs. There was some evidence supporting the role of warmth (H4d) and morality (H4b) but not assertiveness (H4c, see Table S4).

#### 3.5.4. Liking

Supporting H5a, warmth was a robust mediator of the relationship between GP communication and liking of GPs. There was some evidence supporting the role of competence and, to a lesser extent, morality (H5b) but not assertiveness (see Table S5).

## 4. Discussion

This study examined whether adherence to NICE-recommended communication strategies, in hypothetical scenarios, causes patients to view GPs more positively and influences their willingness to discuss depression. Specifically, using the MAC model (Prestwich et al., 2021), it examined whether these strategies improve perceptions of respect, liking and trust, and tested the psychological mechanisms underpinning these effects (competence, warmth, assertiveness and morality). The results confirm that NICE-recommended communication strategies likely meaningfully enhance GP–patient encounters. Aside from GP 1 (who did not increase respect), all six communication strategies improved perceptions of GPs (respect, liking, trust, competence, morality, warmth) and willingness to discuss depression relative to baseline (supporting H1a to H1f and H1h). Findings regarding assertiveness were mixed with some GPs increasing assertiveness and others not (providing limited support for H1g). The effects of the communication strategies on the outcomes were not moderated by depression history.

These findings support existing literature that emphasise the critical role of communication in shaping the doctor–patient relationship (e.g., Thompson & McCabe, 2012; Riedl & Schüßler, 2017) and highlight the potential value of NICE guidelines in encouraging openness in mental health consultations. Moreover, as with previous research (Prestwich et al., 2025a), the results of the current study suggest that the impact of adhering to each communication recommendation may not be equal. Ensuring enough time for patients to describe and discuss any problems or concerns yielded the most positive changes in GP evaluations for respect (albeit similar to ensuring all discussions take place in settings maintaining patients’ confidentiality, privacy and dignity), liking, warmth and willingness to discuss depression. However, this strategy would likely be one of the most difficult to implement due to the large number of patients that GPs consult with on a daily basis (Pulse, 2024) and the challenges of time constraints (Dugdale et al., 1999; Freedman et al., 2021). It is therefore critical to consider other strategies that were still significant in improving patient perceptions and willingness to discuss depression, while being more feasible within existing time constraints. Several strategies were equally effective in promoting trust (GP 3: providing information, and discussing with patients, about different treatment options and side effects; GP 4: ensuring enough time for patients to describe and discuss problems or concerns; GP 5 ensuring all discussions take place in settings that maintain patients’ confidentiality, privacy, and dignity; GP 6: allowing enough time towards the end of the appointment to summarise conclusions and enable discussion that allows questions and answers). This pattern was similar for competence.

When not focused on mental health and, instead, commitment to following non-specified GP advice or treatment plan, GP trust and to a lesser degree respect, but not liking, has been found to play a role (Prestwich et al., 2025a). In the current study regarding willingness to discuss depression, there was robust evidence supporting the mediating role of liking (supporting H2b) and, to some extent, trust (providing some support for H2c) but less so for respect (providing limited support for H2a). Together, across studies, findings indicate that different types of feelings towards GPs (i.e., respect, liking, trust) might be more or less important depending on the type of consultation and/or outcome. Trust may be more important for following guidance and advice post-consultation, but liking might be more important for encouraging patients to discuss mental health issues during consultations.

The findings that liking and trust are important for increasing willingness to discuss depression are consistent with prior research highlighting the importance of these constructs for encouraging patients to seek treatment and discuss sensitive information (see Hall et al., 2001; Kannan & Veazie, 2014). A patient may highly respect a GP’s credentials or expertise without feeling emotionally comfortable enough to share personal mental health struggles. It is important to also note that, unlike liking and trust, which are heavily influenced by interpersonal warmth and perceived competence, respectively, respect can be rooted in long-standing perceptions of professionalism and expertise or simply due to status (Lalljee et al., 2009). Patients may already hold GPs in high regard based on their medical qualifications and role in healthcare, making fluctuations in respect less impactful on decisions related to self-disclosure. Indeed, at baseline, GPs were respected more than trusted and liked and respect also increased less than trust and liking when adhering to communication guidelines, as found also in prior work (Prestwich et al., 2025a). The relatively smaller impact of adhering to communication guidance on respect may contribute to its limited role as a mediator between communication guidance adherence and willingness to discuss depression. Without manipulating who the evaluated (GP vs. non-GP) and evaluators (patients vs. non-patients) are, it is not clear whether the study findings are unique to patient–GP relations. As noted, GPs are already highly respected and this may have contributed to relatively smaller effects on respect; for individuals who are not GPs and have limited status, the impact of such communication may be stronger.

The findings were somewhat consistent with the MAC model of respect and liking (Prestwich et al., 2021): morality was the most robust mediator for respect (supporting H3b) and competence also played some role (providing some support for H3a); warmth was the most robust mediator for liking (supporting H5a). Competence was a robust mediator of trust (supporting H4a) with some support for morality (H4b) and warmth (H4d). However, some findings did not neatly align with the MAC model although, interestingly, they did align with the results of a recent test of the MAC model in a healthcare context (Prestwich et al., 2025a).

First, competence emerged as a significant mediator of liking. Patients may have interpreted competence in terms of interpersonal competence and care, and this might be particularly true when discussing mental health rather than physical health. Second, morality was a less reliable mediator than predicted (competence was more consistent for respect; warmth was more consistent for liking). At least compared to changes in warmth (and to a lesser degree, competence), however, changes in morality were much more limited. Third, there was some support that warmth mediated effects on respect, particularly when not controlling for liking. There have been similar findings in the past. For example, Oleszkiewicz and Lachowicz-Tabaczek (2016) reported that warmth shown by super-ordinates (which equate to GPs in the current study) compared to subordinates (equating to patients in the current study) led to greater levels of respect (as well as trust and liking). In light of such findings, the MAC model may need revising to account for the potential moderating role of status of the evaluated person. Fourth, assertiveness did not mediate the effects of the communication guidelines on respect. However, assertiveness must not be excessive as this can damage the doctor–patient relationship (Gutgeld-Dror et al., 2024). That is unlikely to be the case here, however, given the changes in assertiveness were modest compared to changes in competence, warmth and morality. The strategies used may have portrayed collaborative, patient-centred dialogue, which reflect soft interpersonal skills more than directive or assertive behaviour. As a result, participants may not have perceived these scenarios generally as assertive, leading to low variability in assertiveness ratings and reduced potential for it to mediate outcomes.

The current study utilised the MAC model in its development of hypotheses, selection of measures and analyses to test the impact of different types of communication on respect, liking and trust of GPs and the role of these factors for willingness to discuss sensitive topics. Other models are relevant for examining these relationships. For instance, Advice Response Theory (ART; Feng & MacGeorge, 2010) argues that characteristics of the source (in this case, the GP) such as trustworthiness and how much they are liked *and* message content (e.g., politeness, feasibility, perceived efficacy of the advice) can influence outcomes including perceived message quality, how much the advice will help the recipient cope, and their intentions to implement the advice. These relations can be moderated by situational factors such as severity of the issue. According to the model, the impact of source characteristics on outcomes is likely weaker than message content: the role of source characteristics is more indirect such that positive perceptions of the source can increase positive evaluations of the message (e.g., how polite it is seen to be, how efficacious it likely is) that, in turn, predicts outcomes such as likelihood of implementing the advice. Communication Accommodation Theory (CAT; e.g., Gallois et al., 2006) originating from Speech Accommodation Theory (Giles, 1973) argues that individuals can use convergence (adapting their communication behaviour to more closely resemble that of the recipient), divergence (to accentuate such differences) or maintenance (sticking with one’s own style regardless) with convergence being used to create positive evaluations and identities. Such theories are important in highlighting the broader inputs and processes, the bidirectional relationships between them, and their dynamic nature beyond just considering the effect of specific communication strategies on how much a GP (or source more generally) is trusted, liked and respected. The current study, and the MAC model more generally, complements theories such as ART and CAT by bringing to the fore more explicitly the concept of respect alongside the pathways through which this and trust and liking might emerge. In addition, while ART focuses on the effects of source characteristics and message content on various outcomes, the current study adds value by considering the impact of message content on source characteristics—specifically, the extent to which GPs are respected, trusted and liked (as well as how much they are seen as competent, moral, assertive and warm). Future research could present tests of the MAC model alongside models such as ART and CAT in real-life or simulated interactions, for example, to ascertain the value of such combined approaches in complex scenarios.

### 4.1. Strengths and Limitations

The study offers novel insights by being the first study to test the MAC model in the context of primary mental health care and provides an original test of the NICE communication strategies recommended for mental health contexts (NICE, 2011). By focusing specifically on primary mental health care, the current research offers more targeted insights into communication strategies that are potentially effective in promoting willingness to discuss depression. Moreover, the design enabled ‘head-to-head’ comparisons of the potential impact of six different communication recommendations, considered understudied factors that might contribute to patients’ decisions to discuss depression (respect, liking and trust of a GP as well as perceptions of their competence, morality, assertiveness and warmth) and adopted a theory-informed approach to help understand how changes in respect, liking, trust as well as willingness to discuss depression may emerge.

However, the study is not without its limitations. First, all communication scenarios were hypothetical and brief, focused on the specific phrasing of the recommendation rather than capturing the full complexity of GP–patient interactions, thus limiting ecological validity. Since responses were not based on direct engagement with a healthcare provider, participants may not have fully experienced the emotional and relational nuances (for example, convergence as described in CAT) that influence perceptions of respect, trust and willingness to discuss depression. Second, the use of self-report to assess willingness to discuss depression could have introduced social desirability bias, where participants may have reported a greater willingness to disclose depression than they would in a real GP consultation. Self-report measures rely on participants’ introspection and honesty, but actual behaviours may differ in clinical settings due to stigma, discomfort or concerns about confidentiality (Gulliver et al., 2010; Clement et al., 2015). Third, while the study tested six key communication strategies, GP–patient interactions involve additional factors such as non-verbal behaviours and communication, which were not assessed. Fourth, all constructs were measured with single-item scales. This reduced participant burden and aligned with the practical constraints of the study. The questionnaire item assessing depression history did not differentiate between clinical diagnoses, severity, recency or treatment history. Furthermore, individuals with past experiences of depression may have greater sensitivity to how they are treated by healthcare professionals; for example, experiences of stigma associated with mental health conditions can influence expectations of care and shape healthcare interactions (Clement et al., 2015). However, such nuances may not be captured when participants are rating single-item measures and presented with abstract scenarios rather than engaging in face-to-face clinical interactions. Fifth, manipulation checks were not incorporated into the study to determine, for example, that participants perceived GP 2 as more likely to explain clinical and unfamiliar language better than the other GPs. However, the scenarios used were very short and direct. Using GP 2 as an example, given the description (‘GP 2 always explains any clinical or unfamiliar language’), it is unlikely that other GPs would have been rated higher on their ability to explain clinical or unfamiliar language. Moreover, even if there was an alternative explanation for the observed effect, a successful manipulation check still would not rule out the other plausible explanation (Sigall & Mills, 1998). Lastly, the study was UK-based and specific to the UK’s National Health Service (NHS), where GP consultations are often time-limited due to high patient demand (Pulse, 2024). This may not be the same for cultures with differing healthcare systems, for example, insurance-based systems and private healthcare. Previous work has also indicated that the relationship between specific aspects of consultations (such as having sufficient time and being involved in care decisions) and trust differs depending on factors such as age and ethnicity (Croker et al., 2013).

### 4.2. Conclusions

This study highlights how adherence to NICE-recommended communication strategies significantly enhances patients’ perceptions of GPs, while also improving willingness to discuss depression. Among the strategies examined, ensuring sufficient time for patients to describe and discuss concerns was the most impactful. The findings provide partial support for the MAC model. Where the findings were not neatly aligned (e.g., warmth being important for respect), the findings were consistent with a similar previous study that also considered the effects of communication strategies on evaluations of GPs (Prestwich et al., 2025a) and a study indicating warmth from a superior can impact respect (Oleszkiewicz & Lachowicz-Tabaczek, 2016) suggesting there may be important contextual moderators that should be incorporated within a revised version of the MAC model. Liking and trust consistently mediated willingness to disclose depression, whereas respect played a more limited role. The study contributes both theoretical insights into the interpersonal mechanisms underpinning doctor–patient relationships, and practical guidance for improving communication in primary mental health care. While methodological limitations must be acknowledged, the study lays an important foundation for future research to further explore how, and what types of, communication can strengthen therapeutic relationships in general practice.

## Data Availability

Study materials, data and syntax are available on the OSF (https://osf.io/x3qs7/overview?view_only=91104ec58f8744ec9d2113af67cbc076 (accessed on 6 March 2026)).

## Supplementary Materials

The following supporting information can be downloaded at: https://www.mdpi.com/article/10.3390/bs16050668/s1, Table S1: Means and Standard Deviations of Measures Across Baseline and Following Recommendation Conditions (N = 329); Table S2: Mediation Analyses—Effect of a Hypothetical GP Following Communication Recommendations on Patients’ Willingness to Discuss Depression; Table S3: Mediation Analyses—Effect of a Hypothetical GP Following Communication Recommendations on Patients’ Respect for GPs; Table S4: Mediation Analyses—Effect of a Hypothetical GP Following Communication Recommendations on Patients’ Trust of GPs; Table S5: Mediation Analyses—Effect of a Hypothetical GP Following Communication Recommendations on Patients’ Liking of GPs.

## Abbreviations

The following abbreviations are used in this manuscript:

MAC	Morality–Agency–Communion
NHS	National Health Service
GP	General practitioner
NICE	National Institute for Health and Care Excellence
UK	United Kingdom
ANOVA	Analysis of Variance
