# Supplementary material for: Can Adhering to Clinical Communication Guidelines Promote Trust, Liking and Respect for General Practitioners and Willingness to Discuss Depression? A Theory-Based Test
Source: Behav Sci (Basel). 2026 Apr 28;16(5):668. doi: 10.3390/bs16050668 (PMC13203584; doi:10.3390/bs16050668)
Supplement: Supplementary file 1 [file behavsci-16-00668-s001.zip › behavsci-4238623-supplementary.pdf]

Table S1

Means and Standard Deviations of Measures Across Baseline and Following Recommendation Conditions (N = 329)

Measure	GPb		GP 1		GP 2		GP3		GP 4		GP5		GP6		$F(6, 1968)$	η_p^2
	M	*SD*	*M*	*SD*	*M*	*SD*	*M*	*SD*	*M*	*SD*	*M*	*SD*	*M*	*SD*		
Respect	5.77[1]	1.08	5.77[1]	1.17	5.97[2]	0.97	6.16[3]	0.81	6.28[4]	0.78	6.22[3,4]	0.81	6.16[3]	0.81	37.92	.10
Trust	5.35[1]	1.16	5.49[1]	1.17	5.76[2]	1.08	6.09[3]	0.88	6.05[3]	0.92	6.07[3]	0.90	5.99[3]	0.91	70.76	.18
Liking	5.14[1]	1.20	5.52[2]	1.19	5.65[2]	1.10	5.83[3]	1.00	6.09[4]	0.94	5.82[3]	1.00	5.93[3]	0.92	63.04	.16
Willingness to discuss	5.15[1]	1.48	5.41[2]	1.33	5.54[2]	1.29	5.75[3]	1.16	5.99[4]	1.06	5.83[3]	1.14	5.81[3]	1.14	56.61	.15
Competent	5.31[1]	1.17	5.49[1]	1.14	5.94[2]	0.97	6.12[3]	0.88	6.02[2,3]	0.92	5.95[2]	0.92	6.02[2,3]	0.91	71.96	.18
Assertive	5.05[2,3]	1.08	4.84[1]	1.26	5.12[2,3]	1.16	5.20[2]	1.18	5.05[3]	1.13	5.19[2]	1.13	5.23[2]	1.15	11.83	.04
Moral	5.29[1]	1.10	5.64[2]	1.13	5.60[2]	1.09	5.91[3,4]	0.99	5.84[3]	1.00	6.03[4]	0.98	5.81[3]	0.98	46.37	.12
Warm	4.51[1]	1.20	5.55[3,4]	1.20	5.29[2]	1.17	5.44[2,3]	1.07	5.84[5]	1.06	5.49[3,6]	1.08	5.60[4,6]	1.03	98.35	.23

Note. GPb = Baseline GP; GP 1 = asks the patient how they would like to be addressed; GP 2 = explains clinical/unfamiliar language; GP 3 = provides information about different treatment options & side effects & discusses these; GP 4 = ensures time for patients to describe/discuss problems/concerns; GP 5 = ensures settings that maintain confidentiality/privacy/dignity; GP 6 = enables time to summarize conclusions and allow discussion/questions & answers. Significant differences from Bonferroni post-hoc tests are indicated where GP conditions do not share a superscript for a specific measure. Degrees of freedom reported are the uncorrected degrees of freedom.

Table S2

Mediation Analyses- Effect of a Hypothetical GP Following Communication Recommendations on Patients' Willingness to Discuss Depression

Mediator	GP1 *vs.* Baseline		GP2 *vs.* Baseline		GP3 *vs.* Baseline		GP4 *vs.* Baseline		GP5 *vs.* Baseline		GP6 *vs.* Baseline	
	Effect	*SE*	Effect	*SE*	Effect	*SE*	Effect	*SE*	Effect	*SE*	Effect	*SE*
Single mediator												
Respect	0.00	0.03	-0.10**	0.03	-0.17***	0.04	-0.24***	0.05	-0.19***	0.04	-0.17***	0.04
Trust	-0.07*	0.04	-0.24***	0.04	-0.37***	0.06	-0.31***	0.06	-0.37***	0.06	-0.28***	0.05
Liking	-0.18***	0.04	-0.26***	0.04	-0.25***	0.04	-0.36***	0.06	-0.26***	0.04	-0.31***	0.05
Multiple mediator												
Respect	0.00	0.01	-0.02	0.02	-0.03	0.03	-.0.09*	0.05	-0.04	0.04	-0.04	0.03
Trust	-0.03	0.02	-0.13***	0.05	-0.28***	0.07	-0.17**	0.06	-0.24***	0.07	-0.14**	0.06
Liking	-0.12***	0.04	-0.15***	0.04	-0.09*	0.04	-0.16*	0.06	-0.13**	0.05	-0.18***	0.05

Note. *p < .05, *p < .01, ***p < .001. *SE* are bootstrapped standard errors. GP 1 = asks the patient how they would like to be addressed and uses their preferred name and title; GP 2 = explains clinical and unfamiliar language; GP 3 = discusses treatment options and side effects; GP 4 = ensures enough time to discuss any problems or concerns; GP 5 = ensures discussions in settings that maintain confidentiality, privacy and dignity; GP 6 = allows time at the end to summarize conclusions and enable questions and answers.

Table S3

Mediation Analyses- Effect of a Hypothetical GP Following Communication Recommendations on Patients' Respect for GPs

Mediator	GP1 *vs.* Baseline		GP2 *vs.* Baseline		GP3 *vs.* Baseline		GP4 *vs.* Baseline		GP5 *vs.* Baseline		GP6 *vs.* Baseline	
	Effect	*SE*	Effect	*SE*	Effect	*SE*	Effect	*SE*	Effect	*SE*	Effect	*SE*
Single mediator												
Warmth	-0.42***	0.06	-0.29***	0.05	-0.35***	0.05	-0.41***	0.06	-0.33***	0.06	-0.39***	0.06
Competence	-0.09**	0.03	-0.31***	0.05	-0.36***	0.05	-0.31***	0.05	-0.23***	0.04	-0.31***	0.05
Morality	-0.18***	0.04	-0.13***	0.03	-0.29***	0.05	-0.26***	0.04	-0.30***	0.05	-0.24***	0.04
Assertive	0.03*	0.02	-0.01	0.01	-0.01	0.01	-0.00	0.01	-0.01	0.01	-0.01	0.01
Multiple mediator												
Warmth	-0.21***	0.06	-0.15***	0.04	-0.19***	0.05	-0.16**	0.05	-0.18***	0.05	-0.20***	0.06
Competence	-0.03*	0.02	-0.20***	0.05	-0.18***	0.05	-0.18***	0.04	-0.11***	0.04	-0.15***	0.05
Morality	-0.09***	0.03	-0.06**	0.02	-0.12**	0.04	-0.14***	0.03	-0.15***	0.05	-0.11***	0.04
Assertive	0.01	0.01	0.00	0.00	0.00	0.01	0.00	0.00	-0.00	0.01	-0.00	0.01
Multiple mediator												
Warmth	-0.06	0.06	-0.07	0.04	-0.10*	0.04	-0.05	0.05	-0.09*	0.05	-0.12*	0.05

Competence	-0.02	0.02	-0.15***	0.05	-0.11**	0.05	-0.14***	0.04	-0.07*	0.04	-0.09*	0.05
Morality	-0.05*	0.03	-0.04*	0.02	-0.09*	0.04	-0.12***	0.03	-0.12**	0.04	-0.08**	0.03
Assertive	0.01	0.01	-0.00	0.00	0.00	0.01	0.00	0.00	-0.00	0.01	-0.00	0.01
Liking	-0.15***	0.03	-0.14***	0.03	-0.20***	0.04	-0.22***	0.05	-0.17***	0.04	-0.22***	0.05
Multiple mediator												
Warmth	-0.14**	0.05	-0.07*	0.04	-0.12**	0.04	-0.05	0.05	-0.13**	0.04	-0.09*	0.05
Competence	-0.01	0.01	-0.12***	0.04	-0.07	0.04	-0.13***	0.04	-0.04	0.03	-0.06	0.05
Morality	-0.05*	0.03	-0.03	0.02	-0.08*	0.04	-0.10***	0.03	-0.10**	0.04	-0.07*	0.03
Assertive	0.01	0.01	0.00	0.00	0.01	0.01	0.00	0.00	-0.00	0.01	0.00	0.01
Trust	-0.07*	0.03	-0.16***	0.03	-0.30***	0.05	-0.20***	0.04	-0.29***	0.05	-0.26***	0.05
Multiple mediator												
Warmth	-0.06	0.06	-0.04	0.04	-0.08*	0.04	-0.00	0.05	-0.08	0.05	-0.06	0.05
Competence	-0.01	0.01	-0.10**	0.04	-0.05	0.04	-0.11***	0.04	-0.02	0.03	-0.03	0.04
Morality	-0.04	0.02	-0.03	0.02	-0.07*	0.04	-0.10***	0.03	-0.09*	0.04	-0.06*	0.03
Assertive	0.01	0.01	-0.00	0.00	0.01	0.01	0.00	0.00	-0.00	0.01	0.00	0.01
Liking	-0.09***	0.03	-0.09**	0.03	-0.12***	0.03	-0.15***	0.05	-0.10**	0.04	-0.13***	0.04
Trust	-0.05*	0.02	-0.13***	0.03	-0.25***	0.05	-0.16***	0.04	-0.26***	0.05	-0.22***	0.04

Note. $^*p < .05$, $^*p < .01$, $^{***}p < .001$. *SE* are bootstrapped standard errors. GP 1 = asks the patient how they would like to be addressed and uses their preferred name and title; GP 2 = explains clinical and unfamiliar language; GP 3 = discusses treatment options and side effects; GP 4 = ensures enough time to discuss any problems or concerns; GP 5 = ensures discussions in settings that maintain confidentiality, privacy and dignity; GP 6 = allows time at the end to summarize conclusions and enable questions and answers.

Table S4

Mediation Analyses- Effect of a Hypothetical GP Following Communication Recommendations on Patients' Trust of GPs

Mediator	GP1 *vs.* Baseline		GP2 *vs.* Baseline		GP3 *vs.* Baseline		GP4 *vs.* Baseline		GP5 *vs.* Baseline		GP6 *vs.* Baseline	
	Effect	*SE*	Effect	*SE*	Effect	*SE*	Effect	*SE*	Effect	*SE*	Effect	*SE*
Single mediator												
Warmth	-0.40***	0.06	-0.34***	0.05	-0.38***	0.06	-0.59***	0.07	-0.32***	0.05	-0.49***	0.07
Competence	-0.10**	0.04	-0.34***	0.05	-0.45***	0.06	-0.34***	0.05	-0.28***	0.04	-0.41***	0.05
Morality	-0.18***	0.04	-0.16***	0.04	-0.33***	0.05	-0.28***	0.06	-0.31***	0.05	-0.27***	0.05
Assertive	0.03*	0.02	-0.01	0.01	-0.02	0.02	-0.00	0.01	-0.01	0.01	-0.02	0.01
Multiple mediator												
Warmth	-0.16***	0.05	-0.20***	0.04	-0.17***	0.05	-0.36***	0.08	-0.14**	0.05	-0.25***	0.06
Competence	-0.06**	0.02	-0.19***	0.05	-0.28***	0.06	-0.17***	0.05	-0.19***	0.05	-0.24***	0.05
Morality	-0.08***	0.03	-0.06**	0.03	-0.11**	0.05	-0.10**	0.05	-0.13**	0.05	-0.09**	0.05
Assertive	0.01	0.01	0.00	0.00	-0.01	0.01	0.00	0.00	0.01	0.01	-0.00	0.01
Multiple mediator												
Warmth	-0.08	0.05	-0.14***	0.04	-0.08*	0.04	-0.30***	0.08	-0.07	0.04	-0.17***	0.06

Competence	-0.04*	0.02	-0.11***	0.05	-0.19***	0.05	-0.10**	0.05	-0.14***	0.04	-0.17***	0.05
Morality	-0.04*	0.02	-0.04*	0.02	-0.05	0.05	-0.05	0.05	-0.07	0.05	-0.04	0.04
Assertive	0.00	0.01	0.00	0.00	-0.01	0.01	0.00	0.00	0.01	0.01	-0.00	0.01
Respect	0.00	0.03	-0.08**	0.03	-0.18***	0.04	-0.20***	0.04	-0.18***	0.04	-0.17***	0.03
Multiple mediator												
Warmth	0.01	0.05	-0.10**	0.04	-0.06	0.05	-0.21***	0.08	-0.02	0.05	-0.16***	0.06
Competence	-0.05*	0.02	-0.13***	0.05	-0.20***	0.06	-0.11**	0.05	-0.14***	0.05	-0.16***	0.05
Morality	-0.03	0.02	-0.05*	0.02	-0.07*	0.05	-0.07*	0.05	-0.10**	0.05	-0.05	0.04
Assertive	0.01	0.01	0.00	0.00	-0.01	0.01	0.00	0.00	0.01	0.01	-0.00	0.01
Liking	-0.17***	0.04	-0.17***	0.04	-0.24***	0.04	-0.32***	0.06	-0.20***	0.04	-0.25***	0.05
Multiple mediator												
Warmth	0.04	0.05	-0.08*	0.04	-0.02	0.04	-0.19***	0.08	0.01	0.05	-0.12**	0.06
Competence	-0.04*	0.02	-0.09**	0.05	-0.15***	0.05	-0.07	0.05	-0.12***	0.04	-0.13***	0.05
Morality	-0.02	0.02	-0.03	0.02	-0.04	0.05	-0.03	0.06	-0.06	0.05	-0.03	0.04
Assertive	0.00	0.01	0.00	0.00	-0.01	0.01	0.00	0.00	0.01	0.01	-0.00	0.01
Respect	0.00	0.02	-0.06**	0.02	-0.14***	0.03	-0.15***	0.04	-0.15***	0.04	-0.14***	0.03
Liking	-0.13***	0.03	-0.12***	0.04	-0.16***	0.04	-0.26***	0.06	-0.15***	0.04	-0.17***	0.04

Note. $^{*}p < .05$, $^{*}p < .01$, $^{***}p < .001$. *SE* are bootstrapped standard errors. GP 1 = asks the patient how they would like to be addressed and uses their preferred name and title; GP 2 = explains clinical and unfamiliar language; GP 3 = discusses treatment options and side effects; GP 4 = ensures enough time to discuss any problems or concerns; GP 5 = ensures discussions in settings that maintain confidentiality, privacy and dignity; GP 6 = allows time at the end to summarize conclusions and enable questions and answers.

Table S5

Mediation Analyses- Effect of a Hypothetical GP Following Communication Recommendations on Patients' Liking of GPs

Mediator	GP1 *vs.* Baseline		GP2 *vs.* Baseline		GP3 *vs.* Baseline		GP4 *vs.* Baseline		GP5 *vs.* Baseline		GP6 *vs.* Baseline	
	Effect	*SE*	Effect	*SE*	Effect	*SE*	Effect	*SE*	Effect	*SE*	Effect	*SE*
Single mediator												
Warmth	-0.58***	0.07	-0.43***	0.05	-0.50***	0.06	-0.64***	0.08	-0.51***	0.06	-0.53***	0.07
Competence	-0.10**	0.04	-0.36***	0.05	-0.46***	0.06	-0.34***	0.06	-0.31***	0.04	-0.42***	0.06
Morality	-0.21***	0.04	-0.16***	0.04	-0.37***	0.06	-0.28***	0.05	-0.34***	0.05	-0.30***	0.05
Assertive	-0.03*	0.02	-0.01	0.01	-0.02	0.02	-0.00	0.01	-0.02	0.01	-0.02	0.02
Multiple mediator												
Warmth	-0.37***	0.06	-0.30***	0.05	-0.32***	0.06	-0.45***	0.08	-0.38***	0.06	-0.29***	0.06
Competence	-0.03*	0.02	-0.17***	0.05	-0.24***	0.06	-0.19***	0.05	-0.18***	0.04	-0.24***	0.05
Morality	-0.11***	0.03	-0.06**	0.02	-0.12**	0.04	-0.10**	0.04	-0.09*	0.05	-0.11**	0.04
Assertive	0.00	0.01	0.00	0.01	-0.00	0.01	0.00	0.00	0.00	0.01	0.00	0.01
Multiple mediator												
Warmth	-0.28***	0.06	-0.26***	0.05	-0.24***	0.06	-0.39***	0.08	-0.32***	0.06	-0.22***	0.06

Competence	-0.02	0.01	-0.12**	0.04	-0.17**	0.06	-0.12**	0.05	-0.14***	0.04	-0.19***	0.05
Morality	-0.08**	0.03	-0.04*	0.02	-0.07	0.04	-0.04	0.04	-0.05	0.05	-0.07*	0.03
Assertive	-0.00	0.01	0.00	0.01	-0.00	0.01	0.00	0.00	0.00	0.01	0.00	0.01
Respect	0.00	0.03	0.06**	0.02	-0.15***	0.03	-0.19***	0.04	0.14***	0.03	-0.15***	0.03
Multiple mediator												
Warmth	-0.29***	0.06	-0.23***	0.05	-0.25***	0.06	-0.31***	0.08	-0.33***	0.06	-0.19***	0.06
Competence	-0.00	0.01	-0.11**	0.04	-0.13*	0.05	-0.12**	0.05	-0.11**	0.04	-0.15**	0.05
Morality	-0.07**	0.02	-0.04*	0.02	-0.07	0.04	-0.05	0.05	-0.05	0.05	-0.07*	0.04
Assertive	-0.00	0.01	0.00	0.00	0.00	0.01	0.00	0.00	0.00	0.01	0.00	0.01
Trust	-0.08*	0.03	-0.14***	0.04	-0.30***	0.05	-0.27***	0.05	-0.25***	0.05	-0.25***	0.05
Multiple mediator												
Warmth	-0.26***	0.06	-0.22***	0.05	-0.22***	0.05	-0.29***	0.08	-0.31***	0.06	-0.17**	0.06
Competence	0.00	0.01	-0.09*	0.04	-0.11*	0.05	-0.08*	0.05	-0.10**	0.04	-0.14**	0.05
Morality	-0.06**	0.02	-0.03	0.02	-0.05	0.04	-0.03	0.05	-0.03	0.05	-0.06	0.04
Assertive	-0.00	0.01	0.00	0.00	-0.00	0.01	0.00	0.00	0.00	0.01	0.00	0.01
Respect	0.00	0.02	-0.04*	0.02	-0.10**	0.03	-0.13**	0.04	-0.10**	0.03	-0.11***	0.03
Trust	-0.06*	0.03	-0.11***	0.04	-0.23***	0.05	-0.23***	0.05	-0.18***	0.05	-0.18***	0.05

Note. *$p < .05$, *$p < .01$, ***$p < .001$. *SE* are bootstrapped standard errors. GP 1 = asks the patient how they would like to be addressed and uses their preferred name and title; GP 2 = explains clinical and unfamiliar language; GP 3 = discusses treatment options and side effects; GP 4 = ensures enough time to discuss any problems or concerns; GP 5 = ensures discussions in settings that maintain confidentiality, privacy and dignity; GP 6 = allows time at the end to summarize conclusions and enable questions and answers.
